# *Pseudomonas aeruginosa*-mannose-sensitive hemagglutinin inhibits proliferation and induces apoptosis in a caspase-dependent manner in human bladder cancer cell lines

**DOI:** 10.3892/ol.2013.1201

**Published:** 2013-02-19

**Authors:** YI-PING ZHU, XIAO-JIE BIAN, DING-WEI YE, XU-DONG YAO, SHI-LIN ZHANG, BO DAI, YI-JUN SHEN

**Affiliations:** 1 Department of Urology, Fudan University Shanghai Cancer Center, Shanghai 200032, P.R. China; 2 Department of Oncology, Shanghai Medical College, Fudan University, Shanghai 200032, P.R. China

**Keywords:** apoptosis, bladder cancer, caspase, *Pseudomonas aeruginosa*-mannose-sensitive hemagglutinin vaccine

## Abstract

The aim of the present study was to investigate the effects of *Pseudomonas aeruginosa*-mannose-sensitive hemagglutinin (PA-MSHA) on inhibiting the proliferation of bladder cancer cell lines and to further define its functional mechanisms. T24 and 5637 cells were treated with PA-MSHA at various concentrations and times. Cell proliferation was analyzed using Cell Counting Kit-8 (CCK-8) assays. The cell cycle distribution and apoptosis induced by PA-MSHA were measured by flow cytometry with propidium iodide (PI) and annexin V-fluorescein isothiocyanate (FITC) staining. Western blotting was used to evaluate the expression levels of the apoptosis-related molecules and PI3K-AKT-mTOR signaling pathway proteins. A time- and concentration-dependent cytotoxic effect of PA-MSHA was observed in the T24 and 5637 cells. Flow cytometry with PI and annexin V-FITC staining showed that the various concentrations of PA-MSHA were all able to induce the apoptosis and G_0_-G_1_ cell cycle arrest of the bladder cancer cells. Cleaved caspase-8 and -9 and Fas protein expression levels were markedly associated with an increase in the apoptosis of the bladder cancer cells. The cells stimulated with PA-MSHA also exhibited a downregulation of PI3K-AKT-mTOR signaling. PA-MSHA inhibits proliferation and induces apoptosis in the T24 and 5637 bladder cancer cell lines by modulating caspase family proteins and affecting the cell cycle regulation machinery. The PI3K-AKT-mTOR signaling pathway may be important in the direct anticancer cytotoxic effect of PA-MSHA.

## Introduction

Bladder cancer is a major clinical problem worldwide and the incidence has increased over the last two decades, with ∼80% of the diagnosed tumors classified as non-muscle invasive bladder cancer (NMIBC) (1). Treatment for NMIBC includes transurethral resection (TUR) with or without intravesical instillation therapy. The greatest problem in management is the potential for local recurrence, with the recurrence rate ranging between 50 and 70%. In addition, 10 to 20% of NMIBC may further progress to muscle-invasive disease, thus eventually leading to radical cystectomy and urinary diversion (2,3). In the treatment of advanced-stage bladder cancer, combination chemotherapy has exhibited promising results. However, chemotherapy destroys the immune system of the patient, resulting in serious problems (4). In this context, there is a clear requirement to identify novel therapeutic agents which possess immunoregulatory and anticancer effects in patients with bladder cancer.

Engineered bacteria are able to aid in controlling cancer (5). Bacille Calmette-Guérin (BCG), a live-attenuated strain of *Mycobacterium bovis*, is the most common intravesical therapy for NMIBC (6). A vaccine using *Pseudomonas aeruginosa*-mannose-sensitive hemagglutinin (PA-MSHA) has been shown to increase the antigen presenting function by activating the proliferation and differentiation of dendritic cells by the body (7). The PA-MSHA strain is a peritrichous *P. aeruginosa* strain with MSHA fimbriae established by Professor Xi-ya Mu. Furthermore, PA-MSHA possesses cytotoxic qualities due to the addition of MSHA, which has been shown to have anticarcinogenic activity against human hepatocarcinoma and gastric, nasopharyngeal and breast cancer cells (8–11). In addition, an intravesical instillation of 10 ml PA-MSHA in 62 bladder cancer patients was demonstrated to be effective and well tolerated (12). These findings suggest that the use of PA-MSHA may be beneficial in bladder cancer treatment and that it therefore represents a possible tool in adjuvant therapy modalities.

However, the *in vitro* effects of PA-MSHA on bladder cancer cells remain unclear. The present study was designed to investigate the biological therapeutic potential of PA-MSHA against bladder cancer and to further define the functional mechanisms of PA-MSHA.

## Materials and methods

### Reagents

The PA-MSHA used in the present study were kindly provided by Wanter Biopharma Company (Beijing, China), then scale-diluted and stored at 4°C. Phosphate-buffered saline (PBS; 0.1 M; Gibco, Grand Island, NY, USA) was used as a blank control. The following primary antibodies were all from Cell Signaling Technology (Danvers, MA, USA): anti-caspase-8, anti-cleaved caspase-8, anti-caspase-9, anti-cleaved caspase-9, anti-Fas, anti-ERK (tERK), anti-phospho-ERK (pERK), anti-AKT (tAKT), anti-phospho-AKT (pAKT), anti-MTOR (tMTOR), anti-phospho-MTOR (pMTOR) and anti-β-actin.

### Cells lines and culture conditions

The human bladder cancer cell lines, T24 and 5637, were obtained from the Shanghai Institute of Cell Biology, Chinese Academy of Science (Shanghai, China). The cells were cultured in RPMI-1640 medium supplemented with 10% heat-inactivated fetal bovine serum, penicillin (100 U/ml) and streptomycin (100 mg/l) at 37°C in a humidified atmosphere containing 5% CO_2_.

### Cell growth/viability assay

Cell proliferation was analyzed using Cell Counting Kit-8 (CCK-8; Dojindo Molecular Technologies, Inc., Gaithersburg, MD, USA) assays. The cells were plated in 96-well plates (1×10^4^ cells/well) in a concentration- or time-dependent manner. The following day, the medium was changed and fresh medium and the indicated concentrations of PA-MSHA (10, 5, 2.5, 1 and 0.5×10^9^ bacterial cells per ml) were added; the cells were then incubated at 37°C for a further 0, 1, 2 or 3 days. The inhibition of cell growth was determined 24 h later by a reduction assay as follows: 10 *μ*l of CCK-8 was added per well, then the cells were incubated for an additional 4 h and the absorbance at 450 nm was recorded using a 96-well plate reader (Sunrise Microplate Reader, Tecan US, Inc., Charlotte, NC, USA). The following formula was used: Cell viability (%) = [(As − Ab) / (Ac − Ab)] × 100 (n=6, mean ± SD). As was the mean absorbance of the wells with the various concentrations of the drugs added. Ac was the mean absorbance of the diluent wells and Ab was the mean absorbance of the blank wells (no cells, only RPMI-1640). Six replicate wells were used for each analysis and at least three independent experiments were performed.

### Flow cytometry with annexin-V-fluorescein isothiocyanate (FITC) and propidium iodide (PI) staining

The cells were pretreated with the indicated concentrations of PA-MSHA (1, 2.5 or 5×10^9^/ml) for 24 h and single-cell suspensions containing at least 1×10^6^ cells were created. The cell cycle and apoptotic analyses were performed using flow cytometry as described previously, using a FACScalibur system (Becton Dickinson Biosciences, San Diego, CA, USA) (13). The apoptotic cells were analyzed using quadrant statistics on the PI-negative and annexin V-positive cells. Data for the cell cycle analysis were analyzed using ModFit LTTM software (Verity Software House, Inc., Topsham, ME, USA) to determine the proportion of cells in the G_0_/G_1_, S and G_2_/M fractions of the cell cycle. The mean ± SD was calculated for the cell populations from triplicate data.

### Western blot analysis

Cells were lysed according to the standardized protocol (26). Equal amounts of protein lysate at various concentrations were electrophoresed in SDS-PAGE, followed by electroblotting onto polyvinylidene fluoride (PVDF; Immobion™; Millipore, Bedford, MA, USA), for 1 h at 100 V. The membranes were blocked for 1 h at room temperature or overnight at 4°C in 5% skimmed milk in TBS with 0.1% Tween 20. The blot was incubated with the primary antibody (1:1,000), then incubated with a horseradish-peroxidase conjugated secondary antibody (1:3,000; DAKO, Carpinteria, CA, USA). The chemiluminescent detection of antibody binding was performed using an Enhanced Chemiluminescence (ECL) Detection kit (Amersham Pharmacia Biotech, Uppsala, Sweden) and images were captured using the the FUJIFILM LAS-1000 system (Fujifilm, Tokyo, Japan). To ensure that equal amounts of proteins were loaded, the blot was reprobed with rabbit anti-β-actin monoclonal antibodies (1:1,000). A densitometry analysis was performed using the Quantity One software (Bio Rad, Hercules, CA, USA). The relative protein expression levels were normalized by dividing the level of target proteins by the level of β-actin for each sample.

### Statistical analysis

Statistical analysis was performed using the Statistical Package for the Social Sciences (SPSS) software version 15 for Windows (SPSS Inc, Chicago, IL, USA). The differences between pairs of groups were analyzed by two-tailed Student’s t-tests. Differences between multiple groups were evaluated by one-way analysis of variance (ANOVA). P<0.05 was considered to indicate a statistically significant difference.

## Results

### PA-MSHA inhibits the proliferation of bladder cancer cell lines

**Figure 1:**
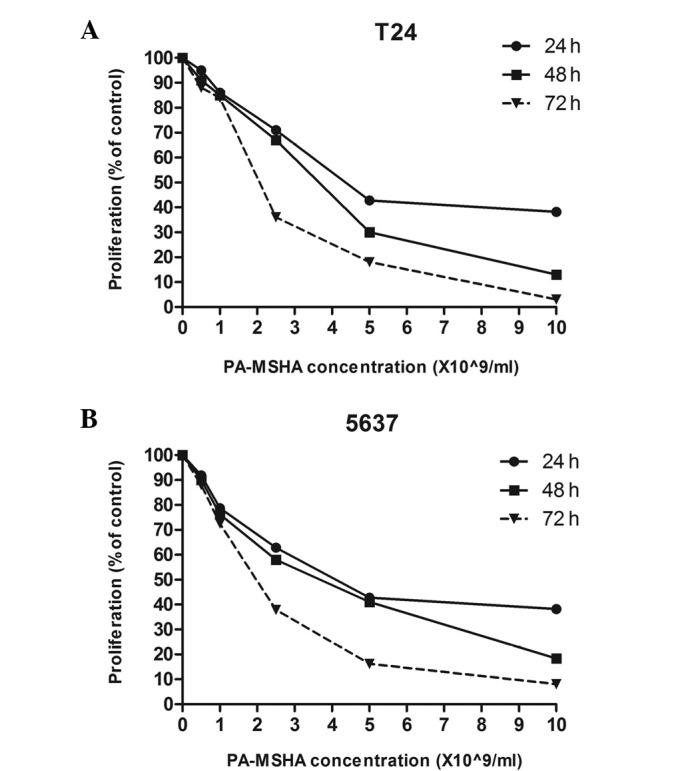
Effect of PA-MSHA on cell proliferation. Values are percentages of the untreated control cells. The data are averages of triplicate results from a representative experiment. P<0.05 for (A) T24 or (B) 5637 cells treated with PA-MSHA vs. controls. PA-MSHA, *Pseudomonas aeruginosa*-mannose-sensitive hemagglutinin.

To determine whether PA-MSHA is a potential bladder cancer treatment agent, its effects on the growth of T24 and 5637 cells were studied. Changes in cell number caused by PA-MSHA were assessed every 24 h using a non-radioactive CCK-8 cell proliferation assay. The 50% percent inhibitory concentrations (IC_50_) are shown in Table I. The exposure of tumor cells to PA-MSHA for up to 72 h had a cumulative effect on T24 and 5637 cell proliferation (Fig. 1A and B, respectively) in a concentration- and time-dependent manner within the same time and concentration range for each cell line.

### PA-MSHA arrests bladder cancer cell lines in the G_0_/G_1_ phase of the cell cycle

**Figure 2:**
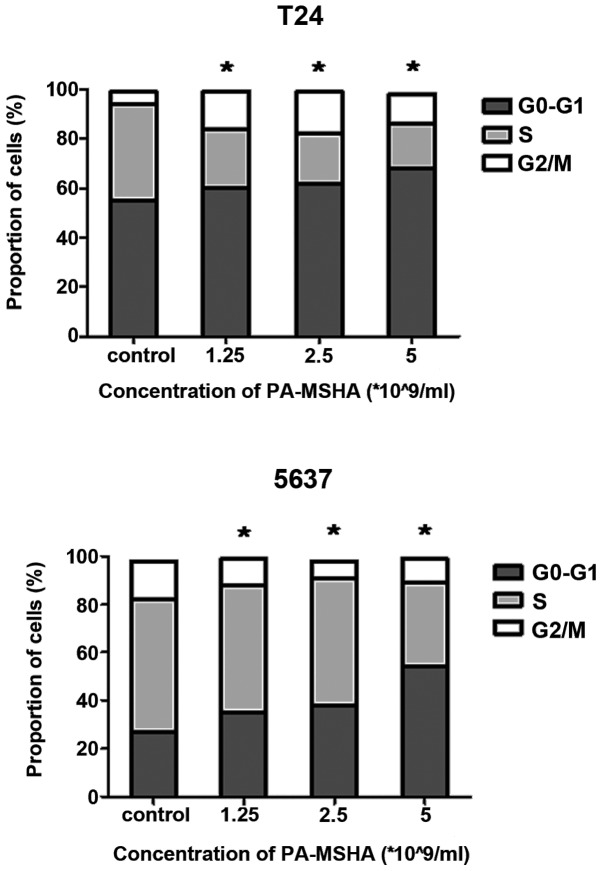
PA-MSHA redistributed cell cycle. The cell cycle distributions of T24 and 5637 in the three phases of the cell cycle are represented by percentages and representative images under these treatment conditions. ^*^P<0.05 for cells treated with PA-MSHA versus controls in all G_0_-G_1_ phases. PA-MSHA, *Pseudomonas aeruginosa*-mannose-sensitive hemagglutinin.

Since PA-MSHA slowed the proliferation of cells, the mechanism by which it exerted these growth-regulatory effects was investigated. The cells were treated with PA-MSHA for 24 h, stained with PI and analyzed using flow cytometry. Treating the T24 and 5637 with increasing concentrations of PA-MSHA (1, 2.5 or 5×10^9^/ml) dose-dependently arrested the cells in the G_0_/G_1_ phase of the cell cycle, thereby decreasing the proportion of cells in the S phase (Fig. 2).

### PA-MSHA induces apoptosis in bladder cancer cell lines

**Figure 3:**
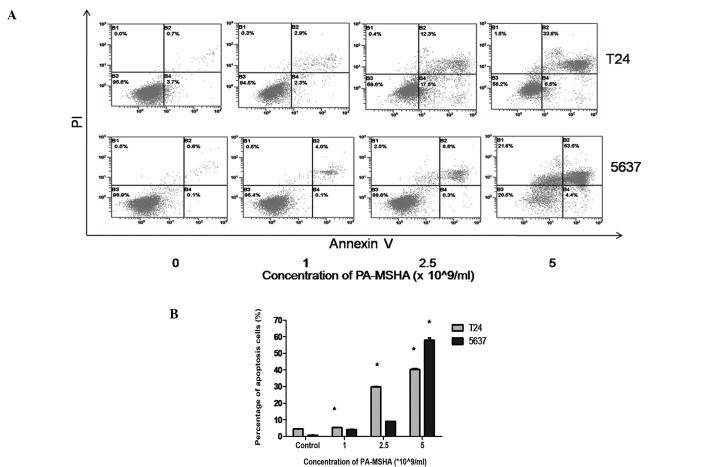
(A) Apoptotic fraction of cells detected by annexin V (x-axis)/PI (y-axis) staining following various treatments. The ratio of apoptotic cells, i.e. the annexin V-positive/PI-negative fraction, was measured in the T24 and 5637 cells in the various concentrations of PA-MSHA (as indicated) with serum-free medium for 24 h. The results are representative of three independent experiments. (B) Percentages of apoptotic cells showing the annexin V-positive/PI-negative fraction. The columns are the mean ± SD of three independent experiments. ^*^P<0.05 for PA-MSHA vs. controls in the T24 and 5637 cells. PA-MSHA, *Pseudomonas aeruginosa*-mannose-sensitive hemagglutinin; PI, propidium iodide.

In the presence of a low dose of PA-MSHA (1×10^9^/ml), slightly elevated numbers of apoptotic cells were detected among the T24 and 5637 cells compared with the controls (Fig. 3A). The apoptotic cell number increased markedly following treatment with high concentrations (2.5 or 5×10^9^/ml) of PA-MSHA (Fig. 3B). It was demonstrated that the T24 and 5637 cells treated with PA-MSHA for 24 h underwent apoptosis in a dose-dependent manner.

### PA-MSHA induces apoptosis using caspase cascade proteins

**Figure 4:**
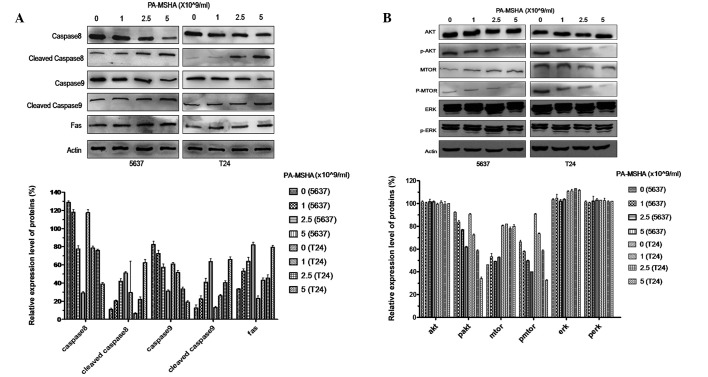
(A) Effect of PA-MSHA on the levels of caspase family proteins in T24 and 5637 cell lines. (B) Effect of PA-MSHA on the levels of AKT, mTOR and ERK and their phosphorylated forms in the T24 and 5637 cell lines. The numeric values of each sample were derived from densitometric analysis and normalized to the expression of β-actin and the bars indicate SD. PA-MSHA, *Pseudomonas aeruginosa*-mannose-sensitive hemagglutinin.

In order to further investigate the mechanisms behind PA-MSHA-induced apoptosis, the T24 and 5637 cells were treated with PA-MSHA to study the activation of caspase-associated proteins. The lysates were analyzed using antibodies against caspase-8 and -9, the cleaved forms of the caspases and Fas protein. As expected, when the T24 and 5637 cells were exposed to PA-MSHA for >24 h, there were dose-dependent losses of procaspase-8 and -9, as well as a concentration-dependent increase in the Fas and cleaved caspase proteins (Fig. 4A), indicating the proteolytic processing of the proenzymes to their active enzyme subunits.

### PA-MSHA inhibits the PI3K-AKT-mTOR signaling pathway

The PI3K-AKT-mTOR signaling pathways are important survival pathways for the regulation of cell survival and proliferation. The present study tested whether treatment with PA-MSHA was able to reduce basal Akt and mTOR phosphorylation. The T24 and 5637 cells were incubated for 48 h in the presence of 1, 2.5 or 5×10^9^/ml PA-MSHA. The treatment with PA-MSHA caused a dose-dependent decrease in pAKT and pmTOR levels but not in pERK, whereas the total levels of AKT, mTOR and ERK remained the same (Fig. 4B). Densitometry indicated that the treatment with 1, 2.5 or 5×10^9^/ml PA-MSHA caused decreases of 17, 32 and 64%, respectively, in phosphorylated Akt and 16, 21 and 68% decreases in phosphorylated mTOR in the T24 cells.

## Discussion

The immunoregulatory effect of PA-MSHA has been well validated. PA-MSHA has been shown to be effective in improving the immune response of patients with several types of cancer and certain other conditions including trauma and infection and chronic diseases such as hepatic fibrosis (14–18). PA-MSHA has also been shown to be effective as a vaccine in plasma phospholipid metabolic profiling and in correcting the ratio of Th2/Th1 cells within the immune organs of mice with IgA nephropathy (19). However, studies on the direct anti-cancer cytotoxic effect of PA-MSHA are limited. Only four studies have demonstrated its anticancer cytotoxicity effect when using hepatocarcinoma, gastric cancer, nasopharyngeal cancer and breast cancer cells (8–11).

The present study is the first systematic attempt to assess the cytotoxic effects of PA-MSHA in bladder cancer cells. It was demonstrated that PA-MSHA is able to inhibit cell proliferation, arrest cells in the G_0_/G_1_ phase and induce cell apoptosis in the T24 and 5637 cells in a dose- and time-dependent manner. In addition, PA-MSHA induced caspase-mediated apoptosis *in vitro* and inhibited the PI3K-AKT-mTOR signaling pathway.

The periods of time and sequence of events from one cell division to the next are collectively referred to as the cell cycle. In the cell cycle analysis, a significant increase in the cell population at the G_0_/G_1_ phase was observed at increased concentrations of PA-MSHA. This result is in agreement with previous findings that showed that PA-MSHA was able to arrest tumor cells in the G_0_/G_1_ phase in breast and nasopharyngeal cancer cells (9,11). Cell cycle checkpoint controls at the G_1_ to S transition prevent the cell cycle from progressing when DNA is damaged. We therefore hypothesize that the G_1_ phase arrest is at least partially explained by the PA-MSHA-induced growth inhibition of the bladder cancer cells. It was also observed that the 24 h IC_50_ doses for the T24 and 5637 cell lines were <4.5×10^9^/ml (Table I), indicating that the tumor cells were sensitive to PA-MSHA.

Caspases have been shown to be activated during apoptosis in numerous cells and are critical in the initiation and execution of apoptosis. Currently, there are two recognized points at which caspases are activated to initiate apoptosis. In the extrinsic pathway, initiator caspase-8 is activated by adapter-mediated recruitment to the death receptor’s cytosolic face following Fas ligation (20). Alternatively, in the intrinsic pathway, initiator caspase-9 is activated following the release of mitochondrial components to form the Apaf complex (21). It has previously been shown that live *P. aeruginosa* is able to induce apoptosis via the extrinsic and intrinsic apoptosis pathways (22,23). The present data suggested that PA-MSHA also acted by triggering the intrinsic and the extrinsic apoptosis pathways, which was consistent with studies performed in breast cancer cell lines (11). However, it is not yet known which pathway is dominant.

The PI3K, AKT and mTOR signal transduction pathways regulate cell survival, proliferation and invasion, all key functions in the progression of bladder cancer (24). In a previous study, the activation of the PI3K-AKT-mTOR pathway correlated with tumor progression and reduced survival in patients with urothelial carcinoma of the urinary bladder (25). In addition, downregulation of the AKT-mTOR signaling pathway predisposed the bladder cancer cells to become apoptotic, indicating that the AKT-mTOR pathway may be an important treatment target for bladder cancer (26). The present study demonstrated that PA-MSHA inhibits the PI3K-AKT-mTOR signaling pathway in a dose-dependent manner. In a previous study in breast cancer cells, Liu *et al* also noted that PA-MSHA inhibited the EGFR and AKT signaling pathways (27). Since targeting the PI3K-AKT-mTOR signaling pathway induces cascade-dependent apoptosis and G_0_/G_1_ cell cycle arrest (28–30), we hypothesize that PA-MSHA exerts it’s antitumor cytotoxic effect by blocking the PI3K-AKT-mTOR signaling pathway. However, further in-depth and detailed experiments are required to verify this theory.

Taken together, the present data demonstrate that PA-MSHA inhibits proliferation and induces apoptosis in the T24 and 5637 bladder cancer lines by modulating caspase family proteins and affecting the cell cycle regulation machinery. The PI3K-AKT-mTOR signaling pathway may have an important role in the direct anticancer cytotoxic effect of PA-MSHA. We propose that PA-MSHA, either alone or in combination with standard therapy, may be a novel strategy for the management of bladder cancer. However, further studies are required to validate the present findings in appropriate animal models.

## Figures and Tables

**Table I:** IC_50_ values of PA-MSHA in various bladder cancer cell lines.

	IC_50_ (×10^9^/ml)		
Cell lines	24 h	48 h	72 h
T24	4.2±0.21	3.6±0.23	2.1±0.19
5637	4.3±0.27	3.8±0.25	2.1±0.22

PA-MSHA, *Pseudomonas aeruginosa*-mannose-sensitive hemagglutinin; IC_50_, 50% inhibitory concentration.
