# [Expression of Concern] *Pseudomonas aeruginosa*-mannose-sensitive hema-gglutinin inhibits proliferation and induces apoptosis in a caspase-dependent manner in human bladder cancer cell lines

**DOI:** 10.3892/ol.2026.15563

**Published:** 2026-03-31

**Authors:** Yi-Ping Zhu, Xiao-Jie Bian, Ding-Wei Ye, Xu-Dong Yao, Shi-Lin Zhang, Bo Dai, Yi-Jun Shen

Oncol Lett 5: 1357–1362, 2013; DOI: 10.3892/ol.2013.1201

Following the publication of this paper, it was drawn to the Editor's attention by a concerned reader that, in Fig. 4B on p. 1360, the western blots shown for the p-AKT and p-MTOR data for the T24 cell line (right-hand gels in the figure part) were strikingly similar, suggesting that these data had been assembled in this figure incorrectly.

The authors were contacted by the Editorial Office to offer an explanation for this apparent anomaly in the presentation of the data in this figure; however, up to this time, no response from them has been forthcoming. Owing to the fact that the Editorial Office has been made aware of potential issues surrounding the scientific integrity of this paper, we are issuing an Expression of Concern to notify readers of this potential problem while the Editorial Office continues to investigate this matter further.

